# Inactivation of photosynthetic cyclic electron transports upregulates photorespiration for compensation of efficient photosynthesis in *Arabidopsis*

**DOI:** 10.3389/fpls.2023.1061434

**Published:** 2023-04-12

**Authors:** Qi Chen, Yixin Lan, Qinghua Li, Mengmeng Kong, Hualing Mi

**Affiliations:** National Key Laboratory of Plant Molecular Genetics, Chinese Academy of Sciences Center for Excellence in Molecular Plant Sciences/Institutes of Plant Physiology and Ecology, Shanghai, China

**Keywords:** photosynthesis, cyclic electron transport around photosystem I, NDH complex, photorespiration, *Arabidopsis*, PGR5-dependent cyclic electron flow, PGRL1

## Abstract

Plants have multiple mechanisms to maintain efficient photosynthesis. Photosynthetic cyclic electron transports around photosystem I (CET), which includes the PGR5/PGRL1 and NDH pathways, and photorespiration play a crucial role in photosynthetic efficiency. However, how these two mechanisms are functionally linked is not clear. In this study, we revealed that photorespiration could compensate for the function of CET in efficient photosynthesis by comparison of the growth phenotypes, photosynthetic properties monitored with chlorophyll fluorescence parameters and photosynthetic oxygen evolution in leaves and photorespiratory activity monitored with the difference of photosynthetic oxygen evolution rate under high and low concentration of oxygen conditions between the deleted mutant PGR5 or PGRL1 under NDH defective background (*pgr5 crr2* or *pgrl1a1b crr2*). Both CET mutants *pgr5 crr2* and *pgrl1a1b crr2* displayed similar suppression effects on photosynthetic capacities of light reaction and growth phenotypes under low light conditions. However, the total CET activity and photosynthetic oxygen evolution of *pgr5 crr2* were evidently lower than those of *pgrl1a1b crr2*, accompanied by the upregulation of photorespiratory activity under low light conditions, resulting in severe suppression of photosynthetic capacities of light reaction and finally photodamaged phenotype under high light or fluctuating light conditions. Based on these findings, we suggest that photorespiration compensates for the loss of CET functions in the regulation of photosynthesis and that coordination of both mechanisms is essential for maintaining the efficient operation of photosynthesis, especially under stressed conditions.

## Introduction

Photosynthesis converts light energy into chemical energy in the form of ATP and NADPH, which drives the two main routes of electron transport: linear electron transport (LET) and cyclic electron transport around photosystem I (CET) (Arnon et al., 1954a; Hill and Bendall, 1960). Electron transport between the two photosystems (photosystem I and II (PSI and PSII, respectively) is mediated by the cytochrome *b_6_f* complex (Cyt*b_6_f*), coupling the translocation of protons across the thylakoid membranes from the stroma to the lumen (*Δ*pH) to drive ATP synthase (Arnon et al., 1954a; Mitchell, 2011). In contrast to LET, CET recycles electrons from the PSI reduction site to the plastoquinone (PQ) pool, coupling the generation of the *Δ*pH *via* Cyt*b_6_f* driving ATP synthase to synthesize ATP without NADPH accumulation (Shikanai, 2007). Two partially redundant pathways of CET operate in angiosperms, both of which are mediated by ferredoxin (Fd)-dependent PQ reduction. The minor pathway is mediated by the chloroplast NADPH dehydrogenase-like (NDH) complex (Munekaga et al., 2004), while the main pathway depends on PGR5/PGRL1 proteins and is likely identical to the historical Arnon’s pathway (Hertle et al., 2013) for its sensitivity to antimycin A (AA) (Arnon et al., 1954a; Arnon et al., 1954b).

CET has puzzled photosynthesis researchers for decades. An important leap occurred when the AA-insensitive pathway was discovered (Endo et al., 1997; Shikanai et al., 1998). The discovery of the NDH pathway was related to the complete sequencing of two plastid genomes, *Nicotiana tabacum* and *Marchantia polymorpha* (Ohyama et al., 1986; Shinozaki et al., 1986). The genomes encode proteins homologous to subunits of mitochondrial NADH dehydrogenase, which is involved in respiratory electron transport in the mitochondria (Matsubayashi et al., 1987). Later, it was found that the reduction of the NDH complex in plants is mediated by Fd (Yamamoto et al., 2011). There is now a consensus that in angiosperms, chloroplast NDH recycles electrons from Fd to PQ and subsequently to PSI through the Cyt*b_6_f*, which is insensitive to AA and is involved in the minor route of electrons in CET (Shikanai, 2007; Peltier et al., 2016). The NDH pathway helps alleviate oxidative stress (Endo et al., 1999; Horvath et al., 2000; Takabayashi et al., 2002; Munné-Bosch et al., 2005; Wang et al., 2006; Yamori et al., 2011).

The AA-sensitive pathway proposed by Arnon has been characterized at the molecular level until the identification of PGR5 and PGRL1 proteins (Munekage et al., 2002; DalCorso et al., 2008). The *Arabidopsis proton gradient regulation 5* (*pgr5*) mutant was discovered in the main pathway of CET when screening the reduced size of non-photochemical quenching (NPQ) of chlorophyll fluorescence (Munekage et al., 2002). *PGRL1* (*pgr5*-like photosynthetic phenotype) genes were discovered based on their co-expression pattern with genes related to photosynthesis and CET perturbation. Additionally, PGRL1 and PGR5 proteins interact physically and functionally in AA-sensitive CET (DalCorso et al., 2008). PGRL1 accepts electrons from Fd in a PGR5-dependent manner and reduces the PQ analog quinone 2,6-dimethyl-*p*-benzoquinone in an AA-sensitive manner (Hertle et al., 2013). PGR5 can operate in CET on its own and is the target of the CET inhibitor AA, but its activity must be modulated by PGRL1 (Ruhle et al., 2021). This is consistent with the fact that a single amino acid substitution of lysine for the third valine residue of mature PGR5 confers resistance to AA (Sugimoto et al., 2013). The PGR5/PGRL1 pathway is essential for achieving a high ATP/NADPH production ratio and for maintaining the appropriate pH range of the thylakoid lumen to induce NPQ and slow down electron transport through the Cyt*b_6_f* during photosynthesis (Shikanai, 2014). PGR5 also plays a key role in adapting to fluctuating light (Suorsa et al., 2012) and is involved in the dominant CET pathway (Shikanai, 2014).

C_4_ plants with high CO_2_ assimilation efficiency in ambient air show higher accumulation of NDH, suggesting that the NDH pathway is important for C_4_ photosynthesis (Takabayashi et al., 2005; Munekage et al., 2010; Nakamura et al., 2013; Ishikawa et al., 2016). Studies on NDH-defective mutants of C_4_ plants showed that the physiological contribution of the NDH pathway is greater in C_4_ photosynthesis than in C_3_ photosynthesis under different light conditions (Ishikawa et al., 2016).

Photorespiration competes with photosynthetic CO_2_ assimilation, which protects the photosynthetic apparatus from photodamage by dissipating excess energy (Osmond et al., 1997). Under limited CO_2_ diffusion conditions, photorespiration lowers the energetic efficiency of photosynthesis in C_3_ plants (Ogren, 1984) to dissipate excess reactive oxygen species (ROS) and energy either directly or indirectly (Voss et al., 2013). Photorespiration is also a major sink for reducing equivalents and ATP to regenerate acceptors for the primary reactions of light reaction (Foyer et al., 2009). When *Arabidopsis* plants grown under high CO_2_ are transferred to ambient air, plants trigger photorespiratory responses due to decreased CO_2_ availability. The enhanced expression of NDH pathway genes (*NDF4* and *NDF6*) under these conditions suggests that CET activation can meet the increased ATP/NADPH demand of photorespiration (Foyer et al., 2012).

In addition, CO_2_ exchange transients confirmed that photorespiration was elevated in maize NDH mutants (Peterson et al., 2016). PGR5 is also required for optimum photosynthesis by sustaining the ATP supply and preventing PSI inactivation, especially with high irradiance or enhanced photorespiratory activity (Munekage et al., 2008). These results indicate that CET could have a close relationship with photorespiration in regulating photosynthesis. However, the functional link between the two processes has not been elucidated.

In this study, we found that completely blocking both the NDH- and PGR5-dependent CET pathways remarkably suppressed photosynthetic capacity accompanied by a significant upregulation of photorespiratory activity. This study discusses the possible functional link between CET pathways and photorespiration and the physiological significance of this relationship.

## Results

### Knockout of *PGR5* and *PGRL1* genes using CRISPR–Cas9

*pgr5-1* was obtained by ethyl methyl sulfone mutagenesis, which likely introduces other mutations into its genome (Munekage et al., 2002). Mutant PGR5 protein (PGR5_G_130_S_) accumulates in *pgr5-1 pgrl2-1* plants (Ruhle et al., 2021). *pgr5-2* mutant has a serine-to-phenylalanine alteration in the middle of mature proteins and accumulates higher levels of PGR5 and PGRL1 proteins than *pgr5-1* mutant (Nakano et al., 2019). To completely knock out *PGR5* (AT2G05620) gene, we constructed a *pgr5* mutant (*pgr5-3*, Col-0 background) using the CRISPR–Cas9 system to insert an adenine in the 243rd base pair downstream of the initiation codon in *Arabidopsis* (**Figure S1A**). We also used the CRISPR–Cas9 system to knock out *PGRL1A* (AT4G22890) (Col-0 background) and *PGRL1B* (AT4G11960) genes (*crr2-2* background and Col-0 background). *PGRL1A* and *PGRL1B* are two highly homologous genes of the *Arabidopsis thaliana*. *pgrl1a-2* has an adenine insertion in the first exon. *pgrl1a-3* has a thymine insertion in the first exon (**Figure S1B**). *pgrl1b-2*, *pgrl1b-3*, *pgrl1b-4*, and *pgrl1b-5* have adenine, cytosine, thymine, and guanine insertions in the first exon, respectively (**Figure S1C**). Insertional mutations in these genes result in premature termination of translation. To confirm the mutation effect, we checked the protein levels of PGR5 and PGRL1 in the mutants. PGR5 protein was hardly detected, and PGRL1 levels significantly decreased to roughly half of wild type (WT) in *pgr5-1* and *pgr5-3* (**Figure S1D**). This indicates that *PGR5* gene is completely knocked out in *pgr5-3* mutant and confirms that the deletion of PGRL1 affects PGR5 protein accumulation (DalCorso et al., 2008). There was no difference in growth phenotype among the mutants and WT under optimal growth conditions (**Figure S1E**). Complementation experiments showed that introducing corresponding genes in the background of *pgr5*, *pgrl1a*, and *pgrl1b* could restore CET activity (**Figure S2**).

### The growth phenotype of *pgrl1a1b crr2* and *pgr5 crr2* under different light conditions

**Figure 1:**
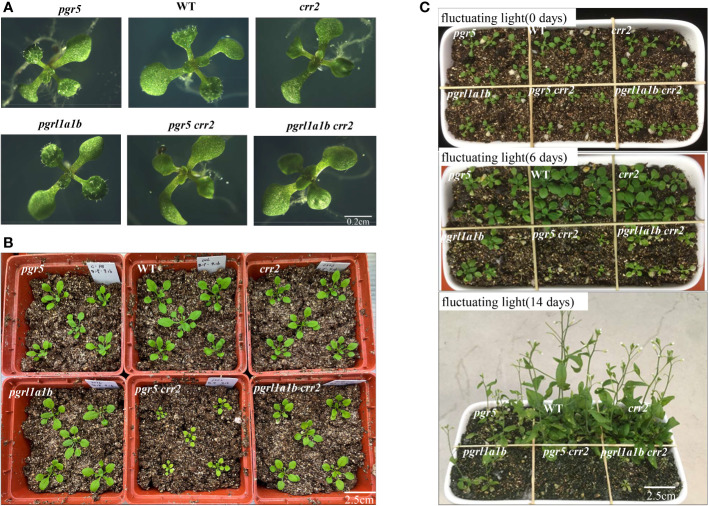
Growth phenotypes of CET mutant lines under different light conditions. **(A)** Plants were grown on agar plates containing Murashige and Skoog (MS) medium, 2% sucrose, and 0.8% agar at a light intensity of 60 μmol m^−2^ s^−1^ at 22°C for 10 days. Bars = 0.2 cm. **(B)** After plants were cultured on the agar plates for 10 days, they were transferred to soil for 2 weeks at a light intensity of 120 μmol m^−2^ s^−1^ at 22°C. Bars = 2.5 cm. **(C)** After the plants grew on the agar plate for 7 days, they were transferred to soil for 5 days under constant light of 80 μmol m^−2^ s^−1^ and shifted to fluctuating light with high light (600 μmol m^−2^ s^−1^) for a 1-min cycle and low light (60 μmol m^−2^ s^−1^) for a 5-min cycle at 22°C. Fluctuating light (0 days): the growth phenotype before the fluctuating light treatment. Fluctuating light (6 days): the growth phenotype in fluctuating light with 6 days. Fluctuating light (14 days): the growth phenotype in fluctuating light with 14 days. Bars = 2.5 cm.

To investigate the functional link of different CET pathways, we first generated mutants that are deficient in the two partially redundant pathways of CET. *pgr5 crr2* double mutant was obtained by crossing *pgr5-3* with the *crr2-2* mutant, and *pgrl1a1b crr2* triple mutant was obtained by crossing *pgrl1a-3* with *pgrl1b-5 crr2-2*. *crr2-2* mutant is impaired in the NDH pathway of CET due to defective in a pentatricopeptide repeat (PPR) protein necessary for the expression of the plastid *ndhB* gene (Hashimoto et al., 2003). We then compared growth phenotypes of mutants with key CET genes knocked out under different light conditions. There was no evident difference in the growth phenotype among single mutants *crr2* and *pgr5*, double mutants *pgrl1a1b* and *pgr5 crr2*, triple mutant *pgrl1a1b crr2*, or WT on Murashige and Skoog (MS) medium supplemented with 2% sucrose and 0.8% agar in a growth chamber with a 16/8-h light/dark cycle and a photosynthetic photon flux density of 60 μmol m^−2^ s^−1^ at 22°C for 10 days (**Figure 1A**). However, after the seedlings were transferred to soil with higher light conditions (120 μmol m^−2^ s^−1^) for 2 weeks, *pgr5 crr2* mutant displayed small and yellowish leaves (**Figure 1B**), while the growth phenotype of triple mutant *pgrl1a1b crr2* behaved like WT, even though the protein level of PGR5 and NDH was similar in both *pgr5 crr2* and *pgrl1a1b crr2* (**Figure S3**). PGR5 was not detected in *pgr5*, *pgrl1a1b*, *pgrl1a1b crr2*, and *pgr5 crr2*, and loss of PGRL1 also blocked PGR5 accumulation. However, NdhJ levels were dramatically lower in *crr2*, *pgr5 crr2*, and *pgrl1a1b crr2*, confirming the deletion of *crr2* in these mutants. Given that PGR5 plays a crucial role in fluctuating light (Suorsa et al., 2012), we also analyzed the growth phenotype of the above mutants under milder fluctuating light conditions (cycles of 5 min low light at 60 μmol photons m^−2^ s^−1^ and 1 min high light at 600 μmol photons m^−2^s^−1^) after 13 days of growth under constant light. There was no significant difference in the growth phenotype before fluctuating light treatment in all mutants and WT (**Figure 1C**, top). However, all the mutants showed visible photodamaged phenotype 6 days after treatment with the fluctuating light: slightly in *crr2*, obviously in *pgr5* and *pgrl1a1b*, severely in *pgrl1a1b crr2*, and most severely in *pgr5 crr2* (**Figure 1C**, middle). This indicates a higher ability to tolerate fluctuating light in *pgrl1a1b crr2* than in *pgr5 crr2*. When the fluctuating light treatment reached 14 days, the photodamaged phenotype of *pgr5* and *pgrl1a1b* was further exacerbated, while lethal phenotype appeared in both *pgrl1a1b crr2* and *pgr5 crr2* (**Figure 1C**, bottom). The results indicate that in addition to the PGR5 pathway, the NDH pathway helps protect against fluctuating light stress. We also analyzed the chloroplast ultrastructure of *pgr5 crr2* and *pgrl1a1b crr2* mutants and WT in 4-week-old leaves grown under 120 μmol m^−2^ s^−1^ light conditions. As shown in **Figure S4**, the WT and *pgrl1a1b crr2* chloroplasts displayed a well-developed membrane system consisting of interconnected stroma lamella and grana lamella, while those of *pgr5 crr2* showed abnormal chloroplast structures, containing few stacking thylakoids and no starch grain (**Figure S4**). The abnormal chloroplasts indicate that PGR5 is essential for photoprotection during chloroplast development. The above findings indicate that *pgrl1a1b crr2* mutant possesses a higher tolerant ability than *pgr5 crr2* mutant under different light conditions.

### CET activity is higher in *pgrl1a1b crr2* than in *pgr5 crr2*

**Figure 2:**
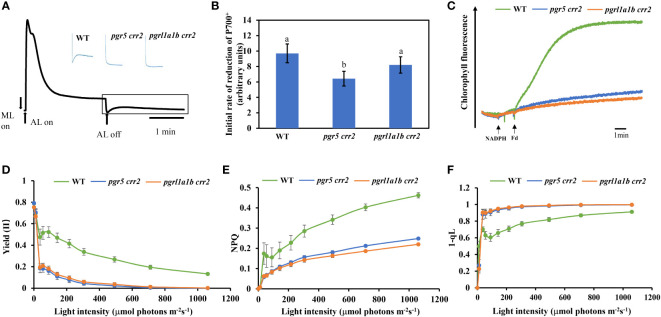
Comparison of CET activity and photosynthetic parameters among CET mutants and wild type (WT). **(A)** Typical kinetics of chlorophyll fluorescence change in WT. Vertical bars indicate the timing of on or off points of white actinic light (AL; 120 μmol photons m^−2^ s^−1^). The part in the rectangle shows the transient increase in chlorophyll fluorescence, which reflects NDH activity. Insets are the traces of different mutants and WT from the boxed area. **(B)** The initial reduction rate of P700^+^ after far-red light. **(C)** Increase in chlorophyll fluorescence induced by the addition of NADPH (0.25 mM) and Fd (5 mM) to osmotically ruptured chloroplasts (20 μg Chl ml^−1^) under the illumination of weak measuring light (1.0 μmol photons m^−2^ s^−1^) in the WT and CET mutants. **(D)** Light curve of the quantum yield of the PSII photochemistry [Yield (II)]. **(E)** Light curve of non-photochemical quenching (NPQ). **(F)** Light curve of redox state of Q_A_ calculated as 1 – qL. Values are means ± SE of five independent measurements. Significant differences (*p* < 0.05) are labeled with different letters on the column. The least difference method (*LSD* method) was used for the difference significance test and multiple ratio comparison. Plants used for these experiments were cultured at a light intensity of 60 μmol m^−2^ s^−1^ at 22°C for 10 days.

To understand the phenotypic differences between *pgrl1a1b crr2* and *pgr5 crr2*, we first compared the CET activity among the seedlings of CET mutants and WT grown under low light (60 μmol m^−2^ s^−1^). As shown in **Figure 2A**, both *pgr5 crr2* and *pgrl1a1b crr2* lost a transient increase in chlorophyll fluorescence after the termination of actinic light illumination as *crr2*, as did other *crr* background mutants, which is similar to the previous results (Munekaga et al., 2004). We further compared the initial dark reduction rate of P700^+^ under far-red light, which reflects CET activity (Mi et al., 1992). The initial re-reduction rate of P700^+^ decreased by approximately 34% in *pgr5 crr2*, but there was no significant difference in *pgrl1a1b crr2*, compared with WT (**Figure 2B**). These results indicate that total CET activity is higher in *pgrl1a1b crr2* than in *pgr5 crr2*. We also checked the electron donation activity of electrons from NADPH to PQ *via* Fd reflected in CET activity, which increased chlorophyll fluorescence by adding NADPH and Fd (Endo et al., 1997). CET activity remarkably decreased in both *pgrl1a1b crr2* and *pgr5 crr2* (**Figure 2C**).

### Impairment of photosynthetic capacities is less serious in *pgrl1a1b crr2* than in *pgr5 crr2* under high light conditions

**Figure 3:**
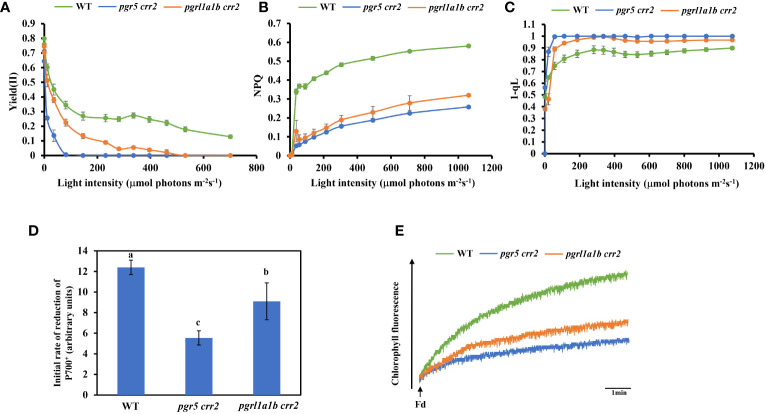
Comparison of chlorophyll fluorescence parameters and CET activity among CET mutants and wild type (WT) grown under high light conditions. **(A)** Light curve of the quantum yield of the PSII photochemistry [Yield (II)]. **(B)** Light curve of non-photochemical quenching (NPQ). **(C)** Light curve of redox state of Q_A_ calculated as 1 − qL. **(D)** The initial reduction rate of P700^+^ after far-red light. **(E)** Increase in chlorophyll fluorescence induced by the addition of NADPH (0.25 mM) and Fd (5 mM) to osmotically ruptured chloroplasts (20 μg Chl ml^−1^) under the illumination of weak measuring light (1.0 μmol photons m^−2^ s^−1^) in the WT and CET mutants. Values are means ± SE of five independent measurements. Significant differences (*p* < 0.05) are labeled with different letters on the column. The least difference method (*LSD* method) was used for the difference significance test and multiple ratio comparison. The plants used for these measurements were cultured on agar plates at 60 μmol m^−2^ s^−1^ at 22°C for 10 days and transferred to the soil at a light intensity of 120 μmol m^−2^ s^−1^ at 22°C for 18 days. Values are means ± SE (n = 5–9) of four independent measurements.

We next compared chlorophyll fluorescence parameters to further examine the photosynthetic performance of the seedlings of CET mutants and WT grown under low light conditions. The light response of quantum yield of the PSII photochemistry [Yield (II)] decreased as light intensity increased; it declined the same faster in both *pgr5 crr2* and *pgrl1a1b crr2*, compared with slower declines observed in WT (**Figure 2D**). NPQ is a chlorophyll fluorescence parameter that reflects the level of thermal dissipation. Increases in the light response of NPQ were detected in WT but were significantly suppressed to a similar degree in *pgr5 crr2* and *pgrl1a1b crr2* (**Figure 2E**), which is consistent with previous observations (Munekage et al., 2002). These results indicate that the light reaction of photosynthesis was suppressed in both mutants under unstressed conditions. The redox state of the PQ pool was also compared among the CET mutants and WT by measuring the chlorophyll parameter of 1 − qL, which reflects the reduction state of Q_A_ in PQ pools (Kramer et al., 2004). The result shows that the light-dependent reduction of Q_A_ increased to a similar degree in both *pgr5 crr2* and *pgrl1a1b crr2* grown under low light conditions (**Figure 2E**), indicating that inactivation of CET causes over-reduction of inter-photosystem electron carriers, such as PQ pool and Cyt*b_6_f*. To investigate the role of the higher CET activity in *pgrl1a1b crr2*, we compared the photosynthetic performance among the mature plants of mutants (*pgr5 crr2* and *pgrl1a1b crr2*) and WT grown under high light conditions. The results show that the decrease in Yield (II) was faster in *pgr5 crr2* (to zero at 80 μmol m^−2^ s^−1^) than in *pgrl1a1b crr2* (to zero at 500 μmol m^−2^ s^−1^) (**Figure 3A**), indicating that *pgrl1a1b crr2* possesses a higher quantum yield of PSII than *pgr5 crr2* under high light conditions. However, NPQ was suppressed to a similar level in response to low light in *pgr5 crr2* and *pgrl1a1b crr2* and slightly lower in *pgr5 crr2* than in *pgrl1a1b crr2* in response to high light (**Figure 3B**). The reduction state of Q_A_ was slightly higher in *pgr5 crr2* than in *pgrl1a1b crr2*, but the difference was not significant. We also compared CET activities among the mutants and WT grown under high light conditions. Both the initial rate of re-reduction of P700 (**Figure 3D**) and the electron donation activity of electrons from NADPH to PQ *via* Fd (**Figure 3E**) were higher in *pgrl1a1b crr2* than *pgr5 crr2*. These results indicated that the higher stress tolerance of *pgrl1a1b crr2* is attributed to its higher CET activity, compared with *pgr5 crr2*.

### Enhancing photorespiration by impairing CET

**Figure 4:**
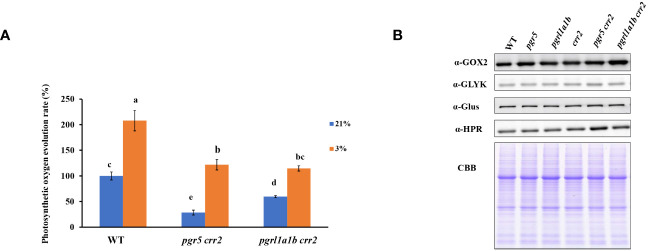
Comparison of photosynthetic oxygen evolution rate and accumulation of key proteins involved in the photorespiratory pathway among the CET mutants and wild type (WT) grown under low light conditions. **(A)** The photosynthetic oxygen evolution rates in CET mutants and WT are under 3% or 21% O_2_. The photosynthetic oxygen evolution rate of WT under 21% was 100% from the value of 5.30 μmol O_2_ gFW^−1^ h^−1^. **(B)** Immunoblotting was performed using antibodies against the GOX2, GLYK, Glus, and HPR in 10-day-old leaves. In the lower panel, a replicate gel stained with Coomassie brilliant blue (CBB) is shown as the loading control. Plants used for these experiments were cultured at a light intensity of 60 μmol m^−2^ s^−1^ at 22°C for 10 days.

The above results demonstrate that PGR5 mutation caused lower CET activity, resulting in a more severe growth phenotype, and low photosynthetic capacities under high light conditions than PGRL1 mutation in *crr2*. Given that previous study has shown that PGR5 plays an important role in photorespiration (Munekage et al., 2008), we compared the photosynthetic rate monitored with photosynthetic oxygen evolution in leaf in the presence of NaHCO_3_ among the CET mutants (*pgr5 crr2* and *pgrl1a1b crr2*) and WT under high oxygen (21% O_2_) conditions favorable to photorespiration and low oxygen (3% O_2_) condition that inhibits photorespiration. We first carried out the experiments using seedlings grown under low light conditions. The photosynthetic oxygen evolution rate decreased significantly in *pgrl1a1b crr2* and much more severely in *pgr5 crr2* when compared with WT at 21% O_2_ (**Figure 4A**), indicating that CET plays an important role in photosynthetic efficiency. However, the rate of oxygen evolution increased approximately twofold in both *pgrl1a1b crr2* and WT and fourfold in *pgr5 crr2* at 3% O_2_ (**Figure 4A**), indicating that *pgr5 crr2* has higher photorespiratory activity than both *pgrl1a1b crr2* and WT. To see whether the upregulation of photorespiratory activity is related to the change in protein level, we compared the amount of several key proteins involved in the photorespiratory pathway. **Figure 4B** shows that there is no evident difference among the CET mutants and WT, suggesting that the upregulation of photorespiratory activity in *pgr5 crr2* is not caused by the increased abundance of key proteins involved in the photorespiration pathway.

**Figure 5:**
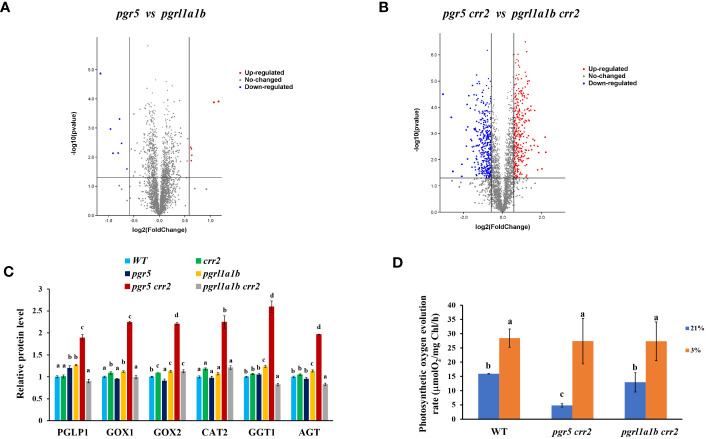
Proteome differences and photosynthetic oxygen evolution between *pgr5 crr2* and *pgrl1a1b crr*2 grown under high light conditions. **(A)** Volcano maps of differentially chloroplast-associated proteins in *pgr5 vs. pgrl1a1b*; there were 7 downregulated proteins and approximately 6 upregulated proteins. **(B)** Volcano maps of proteins in biological differences in *pgr5 crr2 vs. pgrl1a1b crr2*; there were 400 downregulated proteins and approximately 326 upregulated proteins. Red and green spots represent differentially expressed proteins; red spots represent upregulated proteins, and blue spots represent downregulated proteins. **(C)** Comparison of key proteins involved in the photorespiratory pathway and photosynthetic oxygen evolution rate among the CET mutants and wild type (WT). The level of key proteins involved in photorespiration. PGLP1, phosphoglycolate phosphatase 1; GOX1, glycolate oxidase 1; GOX2, glycolate oxidase 2; CAT2, peroxisomal catalase 2; GGT1, glutamate–glyoxylate aminotransferase; AGT, alanine–glyoxylate aminotransferase. There is a significant difference (*p* < 0.05) labeled with different letters on the column. The least difference method (*LSD* method) was used for the difference significance test and multiple ratio comparison. **(D)** The photosynthetic oxygen evolution rates in CET mutants and WT are under 3% or 21% O_2_. Plants for analysis were cultured on agar plates at 60 μmol m^−2^ s^−1^ at 22°C for 10 days and then transferred to soil with a 16/8-h light/dark cycle and a photosynthetic photon flux density of 120 μmol m^−2^ s^−1^ at 22°C for 18 days. Values are means ± SE (n = 5–9) of four independent measurements.

To obtain more comprehensive information on these mutants at the protein level, we performed a proteomic analysis of the CET mutant lines and WT grown under high light conditions. The results show that approximately seven proteins were downregulated and approximately six proteins were upregulated in *pgr5* when compared with *pgrl1a1b* (**Figure 5A**), indicating that *pgr5* and *pgrl1a1b* have similar changes in chloroplast proteome, which strongly correlates with their photosynthesis performances. However, under *crr2* background mutation, both the downregulation and upregulation proteins significantly increased to 400 by approximately 57-fold and 326 by approximately 54-fold, respectively (**Figure 5B**). These results indicate that under *crr2* background mutation, *PGR5* mutation greatly changes the proteome. Differentially expressed proteins between *pgr5 crr2* and *pgrl1a1b crr2* were analyzed with Kyoto Encyclopedia of Genes and Genomes (KEGG), indicating that they are involved in photosynthesis; photosynthetic carbon fixation; carbon metabolism; glycine, serine, and threonine metabolism; glyoxylate dicarboxylate metabolism; pyrimidine metabolism; alanine, aspartate, and glutamate metabolism; ribosome; SNARE interactions in vesicular transport; and biosynthesis of amino acids. The proteins involved in the glyoxylate dicarboxylate metabolism pathway and carbon metabolism pathways were significantly changed. Among the significantly changed proteins, we found that the key proteins involved in photorespiration processes, such as phosphoglycolate phosphatase 1, glycolate oxidase 1, glycolate oxidase 2, peroxisomal catalase 2, glutamate–glyoxylate aminotransferase, and alanine–glyoxylate aminotransferase were dramatically upregulated in *pgr5 crr2* (ranging from 86% to 215%) compared to *pgrl1a1b crr2* (**Figure 5C**). The photosynthetic oxygen evolution rate also decreased significantly in *pgrl1a1b crr2* and much more severely in *pgr5 crr2* when compared with WT at 21% O_2_ (**Figure 5D**), the rate of oxygen evolution increased approximately threefold in *pgr5 crr2*, and there was no significant difference among the CET mutants and WT at 3% O_2_ when the plants were grown under high light conditions (**Figure 5D**). These results indicate that the impairment of both PGR5 and NDH upregulates photorespiration.

## Discussion

### Contribution of NDH, PGR5, and PGRL1 to CET

By comparing different CET mutants, we found that they are both interdependent and complementary. Our work supports previous conclusions that the PGR5/PGRL1 pathway is the dominant CET in C3 plants (**Figure 2**). In the case of NDH deficiency, the loss of PGR5 resulted in the lowest photosynthetic performance **(Figures 3A, B**, **4A**, **5D**) and the poorest tolerance to high light conditions when compared with PGRL1 loss in the same mutation background (**Figure 3**). These results indicate that PGR5 was more important than PGRL1 in the PGR5/PGRL1 pathway, providing additional evidence that PGR5 can independently operate in CET in *pgrl1a1b pgrl2-1* plants.

It has been reported that the localization of PGR5 to the thylakoid membrane is dependent on PGRL1 (DalCorso et al., 2008). When PGRL1 is deleted, the accumulation of PGR5 on the thylakoid membrane is affected, which in turn causes PGR5 to degrade, resulting in undetectable PGR5 protein (**Figure S3**). Although PGR5 protein was not detected in either *pgr5 crr2* or *pgrl1a1b crr2*, *pgrl1a1b crr2* has higher photosynthetic activity (**Figures 3**, **4A**) and better growth phenotype under different light conditions (**Figure 1**), compared with *pgr5 crr2*. The difference is attributed to the higher CET activity in *pgrl1a1b crr2* than in *pgr5 crr2* (**Figures 2B**, **3D, E**), which is essential for efficient photosynthesis (Munekaga et al., 2004). The results indicate that PGRL1 deletion is not equivalent to PGR5 deficiency. To confirm this hypothesis, we generated a quadruple mutant *pgr5 pgrl1a1b crr2*. As expected, the growth phenotype of *pgr5 pgrl1a1b crr2* was similar to *pgr5 crr2* under greenhouse conditions (120 μmol m^−2^ s^−1^) (**Figure S5**). We speculate that *pgrl1a1b crr2* may contain a trace amount of PGR5 protein, with *pgrl1a1b crr2* having a similar phenotype with WT and higher CET activity (**Figure 2B**) under optimal conditions.

### Possible relationship between CET and photorespiration

Previous work had found that *PGR5* mutations increased photorespiratory activity (Munekage et al., 2008) and that mutations in the *Ndh-N* or *Ndh-O* of maize increased photorespiration and decreased the carbon assimilation rate under high light and saturated CO_2_ conditions (Peterson et al., 2016). However, the functional link between CET and photorespiration remains to be investigated. In this work, we found that growth under either low light or high light conditions, completely blocking both the PGR5 and NDH pathways, caused upregulation of photorespiratory activity (**Figures 4A**, **5D**), suggesting that the photorespiratory pathway compensates for the role of CET either in the regulation of ATP/NADPH ratio or in photoprotection (Shikanai, 2014). Phenotype analysis for the plants grown under high light conditions revealed that the deletion of PGR5 under *crr2* background caused photo-oxidative phenotype (**Figure 1B**), resulting in dramatic changes in protein levels (**Figure 5B**), implying the importance of both PGR5 and NDH pathways for multiple functions in addition to photosynthesis. The upregulation of the expression of photorespiration-related proteins (**Figure 5C**) in *pgr5 crr2* grown under high light conditions might have resulted from the oxidative stress (**Figure 1B**) caused by low NPQ (**Figure 3B**). It was found that CET with photorespiration cooperatively regulates the redox state of P700 to suppress the over-reduction in PSI under environmental stress conditions (Takagi et al., 2016). The lowest photosynthetic capacity (oxygen evolution activity) caused by the inactivation of both *PGR5* and *CRR2* genes could be due to the high photorespiratory activity (**Figures 4A**, **5D**). The previous studies and our results indicate that the higher the CET activity, the lower the photorespiratory activity. Therefore, the upregulation of photorespiration in *pgr5 crr2* could be the functional complement for CET-dependent photoprotection and regulation of reductive powers for the loss of CET function. The coordination of CET and photorespiration is essential for maintaining the efficient operation of photosynthesis, especially under stressed conditions.

## Materials and methods

### Generation of CET mutants in *Arabidopsis*

We generated knockout mutants of three nuclear genes, *PGR5*, *PGRL1A*, and *PGRL1B*, *via* CRISPR/Cas9 methods as previously described (Mao et al., 2013). The *PGR5*-, *PGRL1A*-, and *PGRL1B*-specific guide RNA expression sequences were introduced into the CRISPR/Cas9 construct using the primers listed in **Table S1**. Hygromycin was then used to screen the above gene-edited plants without T-DNA, while mutants *pgr5*, *pgrl1a*, and *pgrl1b* containing no T-DNA were used in subsequent experiments.

### Complementation of *pgr5*, *pgrl1a*, and *pgrl1b* mutants

To complement *pgr5*, *pgrl1a*, and *pgrl1b*, cDNA was subcloned into the pGWB17 plasmid with Myc-tag under the control of the 35S promoter. The resulting construct was transformed into the *Agrobacterium tumefaciens* GV3101 strain and introduced into *pgr5*, *pgrl1a*, and *pgrl1b* plants as previously described (Weigel and Glazebrook, 2006). Individual transgenic plants were selected based on resistance to 30 mg/L of hygromycin in half-strength MS medium and 0.8% agar. Resistant plants were transferred to the soil and grown in the growth chamber to produce seeds.

### Growth conditions

WT (Col-0) and *Arabidopsis* mutants (*pgr5*, *crr2*, *pgrl1a1b*, *pgr5 crr2*, and *pgrl1a1b crr2*) were grown on an MS medium containing 2% sucrose and 0.8% agar in a growth chamber under a 16/8-h light/dark cycle with a photosynthetic photon flux density of 60 μmol m^−2^ s^−1^ at 22°C for 10 days. For the high light treatment, the 10-day-old seedlings were transferred to soil with a 16/8-h light/dark cycle and a photosynthetic photon flux density of 120 μmol m^−2^ s^−1^ at 22°C. The seeds were incubated in darkness for 3 days at 4°C before sowing to ensure synchronized germination. For fluctuating light treatment, after the 7-day-old plants grown on MS medium were transferred to soil for 5 days at constant light (80 μmol m^−2^ s^−1^), the light condition was changed to fluctuating light with high light (600 μmol m^−2^ s^−1^) for a 1-min cycle and low light (60 μmol m^−2^ s^−1^) for a 5-min cycle at 22°C.

### Antiserum production

cDNAs encoding the mature PGR5 (amino acids 1–134) and PGRL1A proteins (amino acids 60–200) were amplified by PCR and cloned into the pET51b expression vector (Novagen, Darmstadt, Germany). The resulting plasmids were transformed into the *Escherichia coli* strain BL21. The fusion protein was purified on a nickel-nitrilotriacetic acid agarose resin matrix. The collected protein fraction (6 mg) was sent to Shanghai Immune Biotech to produce the antibodies.

### Protein extraction and immunoblotting

For immunoblot analysis, the total protein samples were prepared from seedling leaves. Samples were denatured with sodium dodecyl sulfate (SDS) sample buffer in a boiling water bath for 5 min and separated by SDS–polyacrylamide gel electrophoresis (SDS-PAGE) in a 12.5% or 15% polyacrylamide gel. The proteins were then electrophoretically transferred onto a polyvinylidene difluoride (PVDF) membrane (Millipore, Billerica, MA, USA) and incubated with antibodies against chloroplast proteins. Signals were identified by an ECL plus Western blot detection system (Tanon).

### Transmission electron microscopy

Leaves used for transmission electron microscopy (TEM) were from the plants first grown on MS medium containing 2% sucrose and 0.8% agar in a growth chamber under a 16/8-h light/dark cycle with a photosynthetic photon flux density of 60 μmol m^−2^ s^−1^ at 22°C for 10 days. The seedlings were then transferred to soil with a 16/8-h light/dark cycle and a photosynthetic photon flux density of 120 μmol m^−2^ s^−1^ at 22°C for 3 weeks. Leaf segments were fixed as previously described (Lan et al., 2021) and observed with a transmission electron microscope (Hitachi H-7650, Tokyo, Japan).

### Isolation of chloroplasts

Intact chloroplasts were isolated from 4-week-old leaves and purified at 4°C as previously described (Wang et al., 2006; Xu et al., 2014). The intact chloroplasts were osmotically ruptured to measure Fd and NADPH-dependent PQ reduction as previously described (Munekage et al., 2002).

### Chlorophyll fluorescence and the redox state of P700

The kinetics of chlorophyll (Chl) fluorescence was measured with Dual-PAM-100 or PAM-100 (Heinz Walz, Effeltrich, Germany). A transient post-illumination increase in Chl fluorescence in *Arabidopsis* leaves was measured after the termination of actinic light (120 μmol photons m^−2^ s^−1^ for 2 min) using a PAM 100 as previously described (Kofer et al., 1998).

The Data Acquisition Software installed in a computer connected to Dual-PAM-100 automatically calculates the Chl fluorescence parameters, Yield (II), NPQ, and Yield (I).

Fd-dependent PQ reduction activity in ruptured chloroplasts was detected by increases in Chl fluorescence by adding NADPH (0.25 mM) and Fd (5 mM) under the illumination of weak measuring light (1.0 μmol m^−2^ s^−1^) using a PAM 100, as described by a previous study (Endo et al., 1998).

The redox state of P700 was measured with a PAM chlorophyll fluorometer (Walz, Effeltrich, Germany) equipped with an emitter-detector ED-P700DW-E unit. P700 absorbance changes were monitored by absorbance at 810–830 nm (Schreiber et al., 1986; Klughammer and Schreiber, 1998). An initial reduction rate (0–1 s) of P700^+^ was estimated as CET activity (Wu et al., 2011).

### Quantitative proteome analysis

Col-0 and *Arabidopsis* mutants (*pgr5*, *crr2*, *pgrl1a1b*, *pgr5crr2*, and *pgrl1a1bcrr2*) were grown on an MS medium containing 2% sucrose and 0.8% agar in a growth chamber under a 16/8-h light/dark cycle with a photosynthetic photon flux density of 60 μmol m^−2^ s^−1^ at 22°C for 10 days. We then transferred the seedlings to soil with a 16/8-h light/dark cycle and a photosynthetic photon flux density of 120 μmol m^−2^ s^−1^ at 22°C for 4 weeks. The leaves of plants from three independent biological replicates were homogenized in Rensink extraction buffer (50 mM Tris/HCl pH 7.5, 100 mM NaCl, 0.5% (v/v) TritonX-100, 2 mM of DTT, and protease inhibitor cocktail (Sigma–Aldrich, Darmstadt, Germany). The samples were then sent to the Orizymes Biotechnologies Company (Shanghai, China) for quantitative proteome analysis.

### Measurement of photosynthetic oxygen evolution

Whole leaves were cut from seedlings grown under low light for 10 days. In contrast, functional leaves from the plants grown under high light for 20 days were cut into small fragments (approximately 1 mm wide). The fragments were then stirred into a 1.8-ml suspension containing 50 mM^–1^ of NaHCO_3_ and 50 mM^–1^ of Tris-HCl (pH 7.5) in the thermostated glass vessel of a Clark-type oxygen electrode. The photosynthetic O_2_ evolution was normally detected several minutes after illumination (800 µmol photons m^−2^ s^−1^), as previously described (Wu et al., 2012).

## Data availability statement

Publicly available datasets were analyzed in this study. This data can be found here: NCBI, PGR5 AT2G05620), PGRL1A (AT4G22890), PGRL1B (AT4G11960), and CRR2 (AT3G46790). The mass spectrometry proteomics data have been deposited to the ProteomeXchange Consortium (http://proteomecentral.proteomexchange.org) *via* the iProX partner repository with the dataset identifier PXD034408.

## Author contributions

HM and QC conceived the project. HM, QC, and YL analyzed the data and wrote the paper. QC and YL performed the main experiments, QL and MK performed partial experiments. All authors contributed to the article and approved the submitted version.

## Supplementary material

Supplementary Figure 1 The *PGR5* and *PGRL1* knockout mutants. **(A)** *PGR5* gene showing the location and type of insertion in mutant line used in this study. *pgr5-3* has an adenine insertion in the first exon. **(B)** *PGRL1A* gene showing the location and type of mutations in mutant lines used in this study. *pgrl1a-2* has an adenine insertion in the first exon. *pgrl1a-3* has a thymine insertion in the first exon. **(C)** *PGRL1B* gene showing the location and type of mutations in mutant lines used in this study. *pgrl1b-2*,*3*,*4*,*5* have adenine, cytosine, thymine, and guanine insertions in their first exons, respectively. **(D)** Immunoblot analysis of the accumulation of PGR5 and PGRL1 in wild type (WT) and mutants of *Arabidopsis*. *pgr5-1* is the control mutant with an amino acid substitution from glycine 130 to serine in previous reports (Munekage et al., 2002). **(E)** Growth phenotypes of mutant lines compared to WT. Plants were grown in soil with a 16/8 h light/dark cycle and light density of 120 μmol m^-2^ s^-1^ at 22°C for 20 days. To ensure synchronized germination, seeds were incubated in darkness for 3 d at 4°C before sowing. Bars =2 cm.

Supplementary Figure 2 Complementation of the *pgr5*, *pgrl1a* and *pgrl1b* mutants. **(A)** Typical kinetics of change in chlorophyll fluorescence in WT. Vertical bars indicate the timing of on or off points of white actinic light (AL, 120 μmol photons m^-2^ s^-1^). The part in the rectangle shows the transient increase in chlorophyll fluorescence, which reflects NDH activity. On the right are the magnified traces from the boxed area of WT, *pgr5*, *pgrl1a*, *pgrl1b* and their complemented plants. **(B)** Immunoblotting was performed using antibody against Myc in WT and the different complemented lines.

Supplementary Figure 3 Comparison of protein levels among CET mutants and WT. Immunoblotting was performed using antibodies against the PGR5, PGRL1, and NDH complex subunit (NdhJ) and the Cyt *b_6_f* complex subunit (PetA) in 28-day-old leaves. Each lane was loaded with 40 μg of total protein. In the lower panel, a replicate gel stained with Coomassie brilliant blue (CBB) is shown as the loading control. Plants used for these experiments were cultured at a light intensity of 60 μmol m^-2^ s^-1^ at 22°C for 10 days, then transferred to 120 μmol m^-2^ s^-1^ at 22°C for 3-4 weeks.

Supplementary Figure 4 Chloroplast ultrastructure of *Arabidopsis* WT, *pgr5 crr2* and *pgrl1a1b crr2* mutants. The chloroplast structures were analyzed from the leaves of 4-week-old WT, *pgr5 crr2* and*pgrl1a1b crr2* plants grown in soil with a 16/8 h light/dark cycle and a photosynthetic photon flux density of 120 μmol m^-2^ s^-1^ at 22°C. GT, grana thylakoids; ST, stroma thylakoids; SG, starch grains. Bars =2μm in the upper panel and 0.5 μm in the below panel.

Supplementary Figure 5 The phenotype of the *pgr5 pgrl1a1b crr2* mutant Plants were grown on MS medium for 10 days, then transferred to the soil for 2 weeks with a 16/8 h light/dark cycle and a photosynthetic photon flux density of 120 μmol m^-2^ s^-1^ at 22°C. To ensure synchronized germination, the seeds were incubated in darkness for 3 d at 4°C before sowing. Bars =2.5 cm.
