# Supplementary figures and images for: Inactivation of photosynthetic cyclic electron transports upregulates photorespiration for compensation of efficient photosynthesis in Arabidopsis
Source: Front Plant Sci. 2023 Apr 12;14:1061434. doi: 10.3389/fpls.2023.1061434 (PMC10130413; doi:10.3389/fpls.2023.1061434)

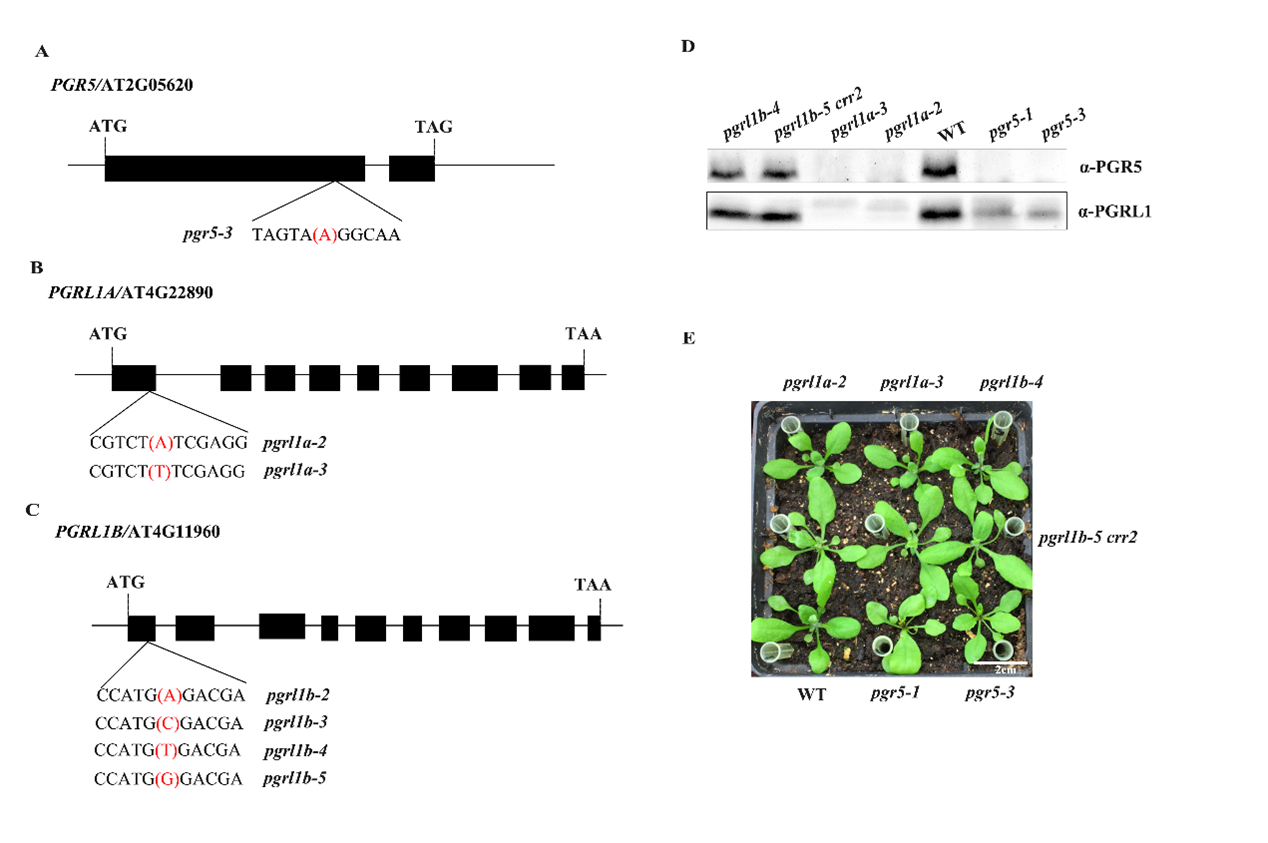

Supplement: Supplementary Figure 1 — The PGR5 and PGRL1 knockout mutants. (A) PGR5 gene showing the location and type of insertion in mutant line used in this study. pgr5-3 has an adenine insertion in the first exon. (B) PGRL1A gene showing the location and type of mutations in mutant lines used in this study. pgrl1a-2 has an adenine insertion in the first exon. pgrl1a-3 has a thymine insertion in the first exon. (C) PGRL1B gene showing the location and type of mutations in mutant lines used in this study. pgrl1b-2,3,4,5 have adenine, cytosine, thymine, and guanine insertions in their first exons, respectively. (D) Immunoblot analysis of the accumulation of PGR5 and PGRL1 in wild type (WT) and mutants of Arabidopsis. pgr5-1 is the control mutant with an amino acid substitution from glycine 130 to serine in previous reports (Munekage et al., 2002). (E) Growth phenotypes of mutant lines compared to WT. Plants were grown in soil with a 16/8 h light/dark cycle and light density of 120 μmol m-2 s-1 at 22°C for 20 days. To ensure synchronized germination, seeds were incubated in darkness for 3 d at 4°C before sowing. Bars =2 cm. [file Image_1.tif]

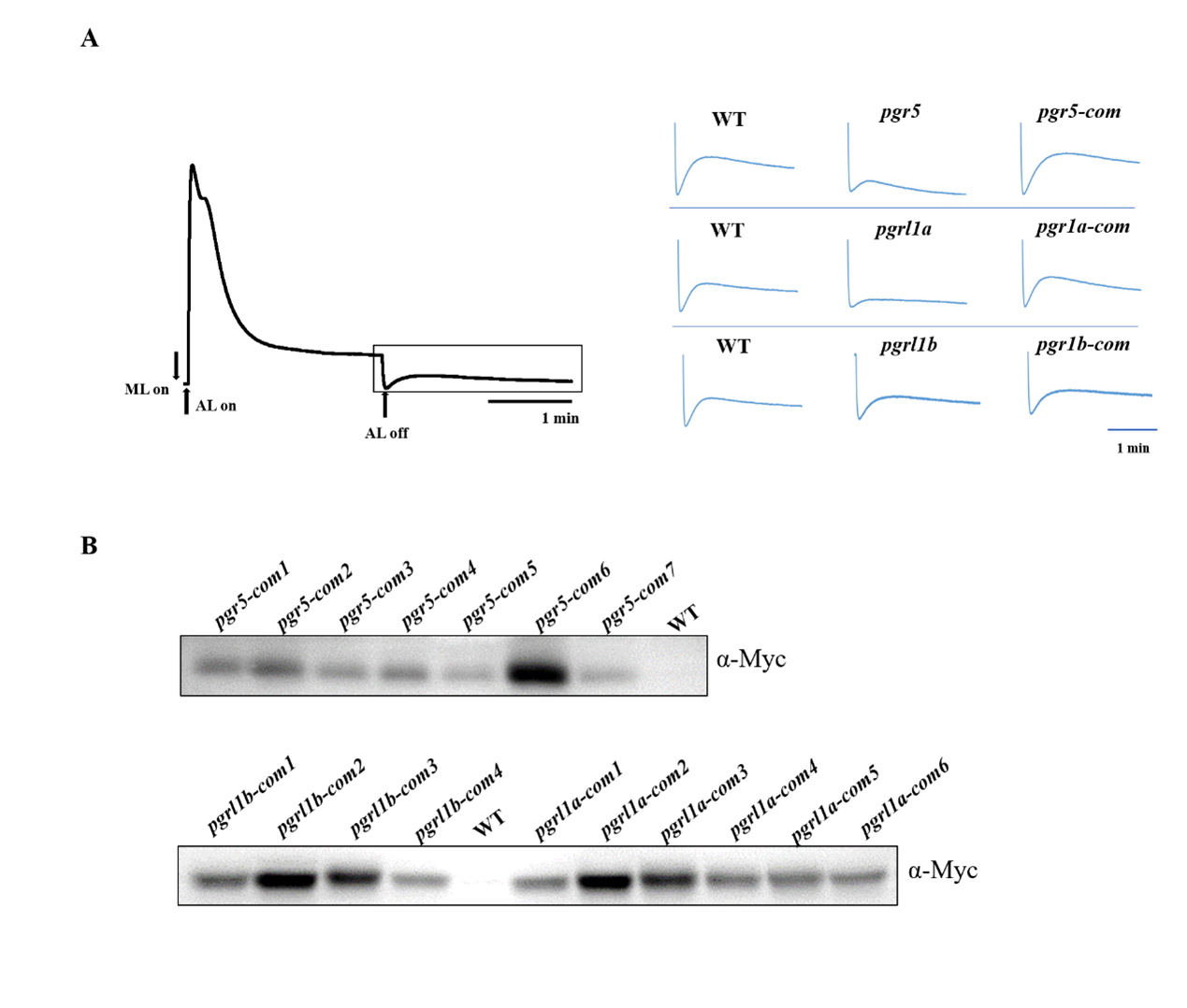

Supplement: Supplementary Figure 2 — Complementation of the pgr5, pgrl1a and pgrl1b mutants. (A) Typical kinetics of change in chlorophyll fluorescence in WT. Vertical bars indicate the timing of on or off points of white actinic light (AL, 120 μmol photons m-2 s-1). The part in the rectangle shows the transient increase in chlorophyll fluorescence, which reflects NDH activity. On the right are the magnified traces from the boxed area of WT, pgr5, pgrl1a, pgrl1b and their complemented plants. (B) Immunoblotting was performed using antibody against Myc in WT and the different complemented lines. [file Image_2.tif]

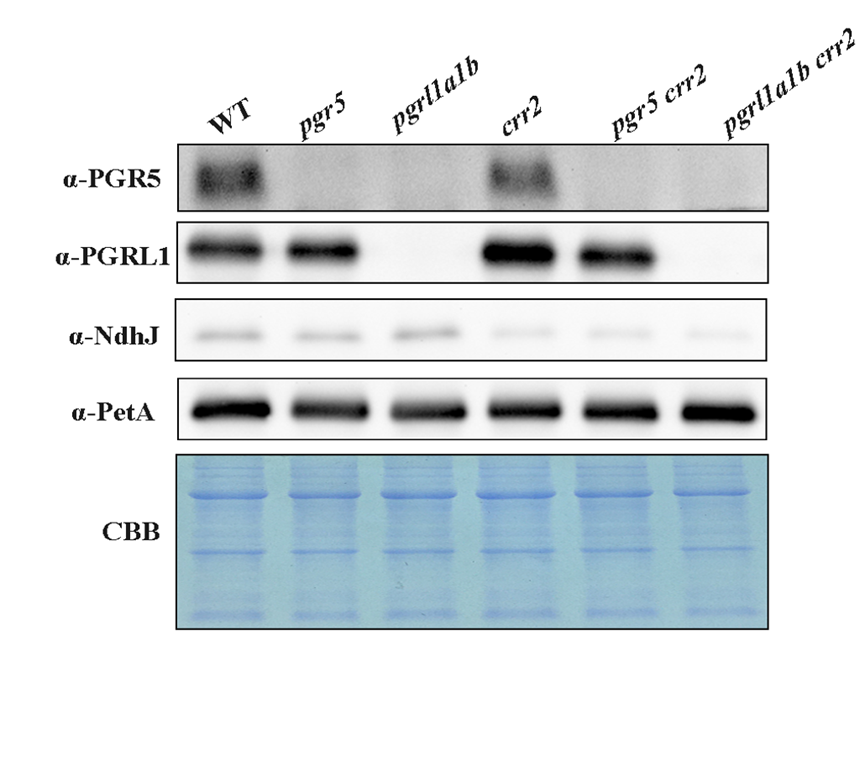

Supplement: Supplementary Figure 3 — Comparison of protein levels among CET mutants and WT. Immunoblotting was performed using antibodies against the PGR5, PGRL1, and NDH complex subunit (NdhJ) and the Cyt b6f complex subunit (PetA) in 28-day-old leaves. Each lane was loaded with 40 μg of total protein. In the lower panel, a replicate gel stained with Coomassie brilliant blue (CBB) is shown as the loading control. Plants used for these experiments were cultured at a light intensity of 60 μmol m-2 s-1 at 22°C for 10 days, then transferred to 120 μmol m-2 s-1 at 22°C for 3-4 weeks. [file Image_3.tif]

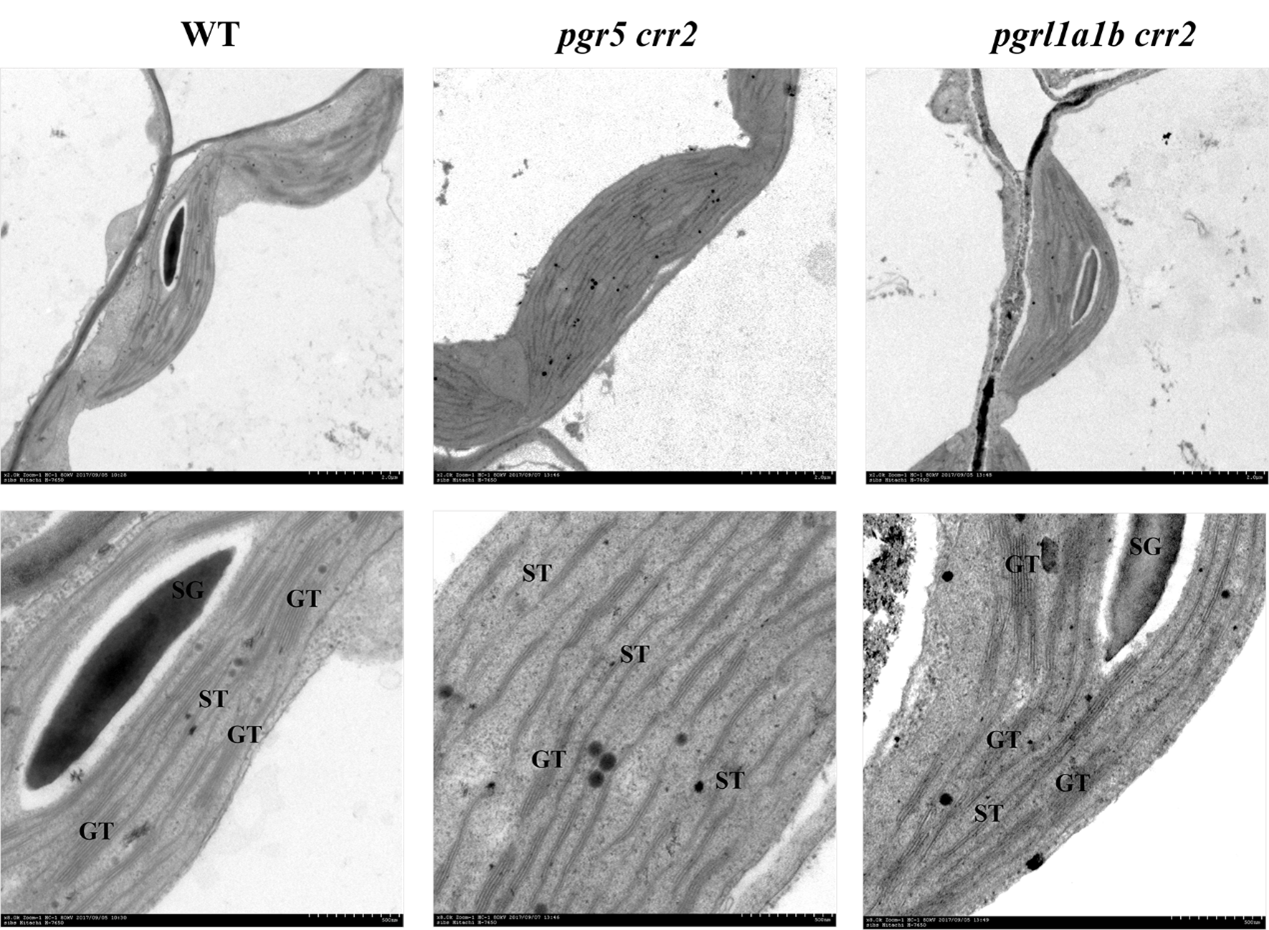

Supplement: Supplementary Figure 4 — Chloroplast ultrastructure of Arabidopsis WT, pgr5 crr2 and pgrl1a1b crr2 mutants. The chloroplast structures were analyzed from the leaves of 4-week-old WT, pgr5 crr2 andpgrl1a1b crr2 plants grown in soil with a 16/8 h light/dark cycle and a photosynthetic photon flux density of 120 μmol m-2 s-1 at 22°C. GT, grana thylakoids; ST, stroma thylakoids; SG, starch grains. Bars =2μm in the upper panel and 0.5 μm in the below panel. [file Image_4.tif]

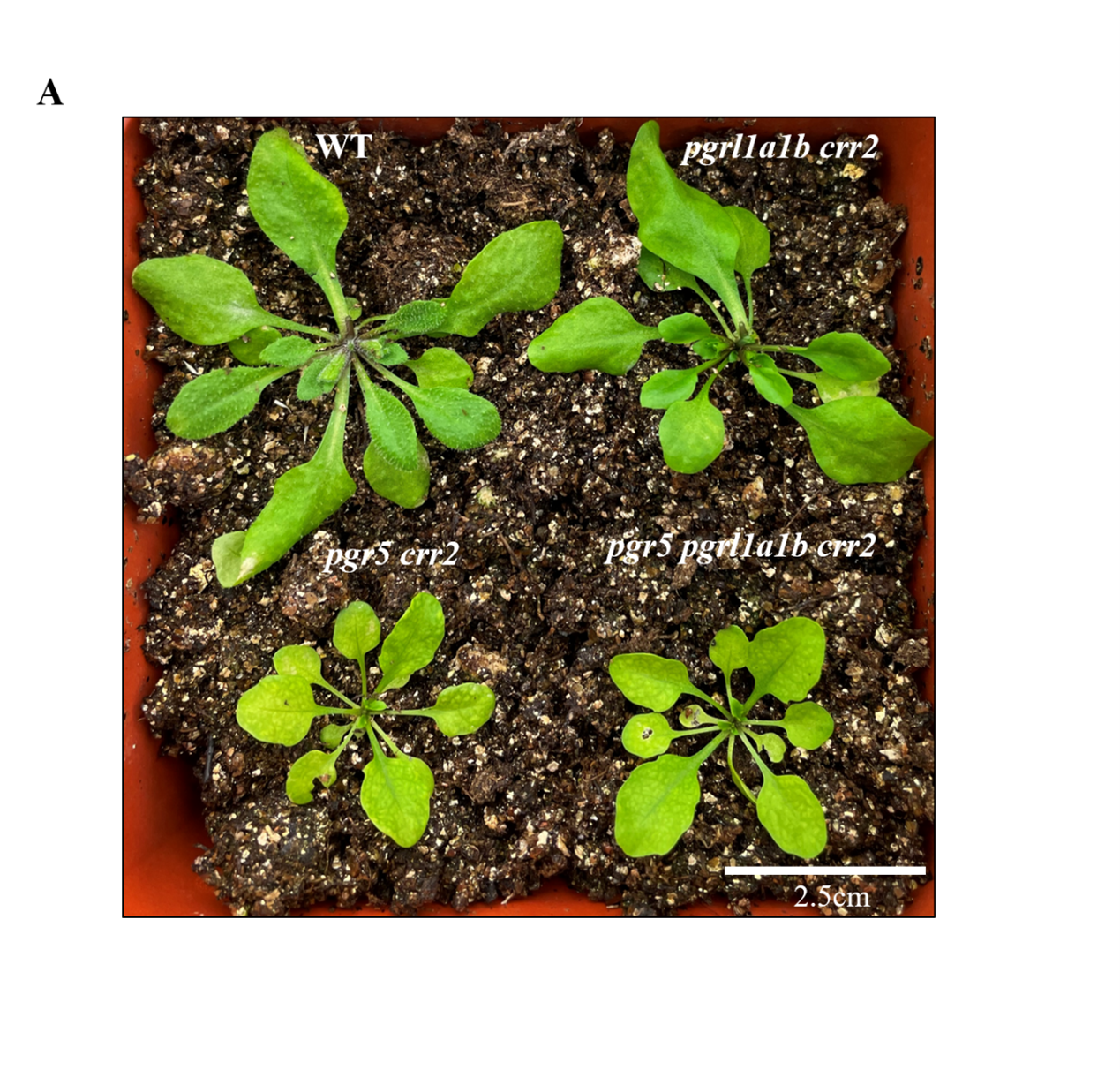

Supplement: Supplementary Figure 5 — The phenotype of the pgr5 pgrl1a1b crr2 mutant Plants were grown on MS medium for 10 days, then transferred to the soil for 2 weeks with a 16/8 h light/dark cycle and a photosynthetic photon flux density of 120 μmol m-2 s-1 at 22°C. To ensure synchronized germination, the seeds were incubated in darkness for 3 d at 4°C before sowing. Bars =2.5 cm. [file Image_5.tif]
