# Supplementary material for: Inactivation of photosynthetic cyclic electron transports upregulates photorespiration for compensation of efficient photosynthesis in Arabidopsis
Source: Front Plant Sci. 2023 Apr 12;14:1061434. doi: 10.3389/fpls.2023.1061434 (PMC10130413; doi:10.3389/fpls.2023.1061434)
Supplement: Supplementary file 6 [file Table_1.docx]

Table 1

| Target gene | Target site (5′-3′) | Oligo 1 (5′-3′) | Oligo 2 (5′-3′) |
| --- | --- | --- | --- |
| *AtPGR5* | TAAACCTCTTCTTGCCTACTAGG | GATTGTAAACCTCTTCTTGCCTACT | AAACAGTAGGCAAGAAGAGGTTTAC |
| *AtPGRl1A* | GTCCATTGAGTCCTCGAAGACGG | GATTGGTCCATTGAGTCCTCGAAGA | AAACTCTTCGAGGACTCAATGGACC |
| *AtPGRl1B* | GAGAGAAATCGATCGTCCATGGG | GATTGGAGAGAAATCGATCGTCCAT | AAACATGGACGATCGATTTCTCTCC |
